# Identification of calcium and integrin-binding protein 1 as a reprogrammed glucose metabolism mediator to restrict immune cell infiltration in the stromal compartment of pancreatic ductal adenocarcinoma

**DOI:** 10.3389/fimmu.2023.1158964

**Published:** 2023-04-28

**Authors:** Junrui Ma, Yue Song, Tongtao Zhuang, Hao Yang, Xiaobao Yang, Juanjuan Zheng, Jiajun Luo, Yihan Xia, Xuefeng Fei, David W. Chan, Di Wu, Peiqing Xu, Peihua Ni, Jing Dai, Dakang Xu, Yiqun Hu

**Affiliations:** ^1^Department of Laboratory Medicine, Ruijin Hospital, Shanghai Jiao Tong University School of Medicine, Shanghai, China; ^2^College of Health Sciences and Technology, Shanghai Jiao Tong University School of Medicine, Shanghai, China; ^3^Medical Technology Department, Qiqihar Medical University, Qiqihar, Heilongjiang, China; ^4^Xiangya School of Medicine, Central South University, Changsha, Hunan, China; ^5^Blood Transfusion Department, Qilu Hospital of Shandong University Dezhou Hospital, Dezhou, Shandong, China; ^6^School of Medicine, The Chinese University of Hong Kong (Shenzhen), Shenzhen, China

**Keywords:** CIB1, metabolic reprogramming, CD8+ T cells, PDAC, glycolysis, immune profiling

## Abstract

An increasing body of evidence has suggested that reprogrammed metabolism plays a critical role in the progression of pancreatic ductal adenocarcinoma (PDAC) by affecting the tumor and stromal cellular components in the tumor microenvironment (TME). By analyzing the KRAS pathway and metabolic pathways, we found that calcium and integrin-binding protein 1 (CIB1) corresponded with upregulation of glucose metabolism pathways and was associated with poor prognosis in patients with PDAC from The Cancer Genome Atlas (TCGA). Elevated CIB1 expression combined with upregulated glycolysis, oxidative phosphorylation (Oxphos), hypoxia pathway activation, and cell cycle promoted PDAC tumor growth and increased tumor cellular com-ponents. Furthermore, we confirmed the mRNA overexpression of CIB1 and co-expression of CIB1 and KRAS mutation in cell lines from the Expression Atlas. Subsequently, immunohistochemistry staining from the Human Protein Atlas (HPA) showed that high expression of CIB1 in tumor cells was associated with an increased tumor compartment and reduced stromal cellular abundance. Furthermore, using multiplexed immunohistochemistry (mIHC), we verified that low stromal abundance was correlated with low infiltration of CD8^+^ PD-1^−^ T cells which led to suppressed anti-tumor immunity. Overall, our findings identify CIB1 as a metabolic pathway-mediated factor for the restriction of immune cell infiltration in the stromal compartment of PDAC and highlight the potential value of CIB1 as a prognostic biomarker involved in metabolic reprogramming and immune modulation.

## 1 Introduction

Pancreatic ductal adenocarcinoma (PDAC) is a gastrointestinal malignancy with a 5-year overall survival rate of only 11% in 2022, largely due to its lethal nature and potent resistance to limited therapeutic options (1, 2). Exocrine neo-plastic changes are observed in the initial stages of PDAC, while pancreatic intraepithelial neoplasms (PanINs) indicate progression toward malignancy. Regarding genetic alternations, mutations in human KRAS have been widely detected and linked to PDAC (3, 4). *In vivo*, genetically engineered animal models have been established to confirm the essential role of oncogenic KRAS in both PDAC initiation and progression (3, 5, 6). Additionally, it has recently been suggested that KRAS-mediated metabolic reprogramming acts as an accelerator in the progression of PDAC (7).

Distinct cellular metabolism is under regulation by numerous factors. KRAS, a GTPase family member, is mutated at a high frequency in pancreatic cancer and is permanently activated to continuously stimulate downstream effectors, notably PI3K and RAF (3, 7). Consequently, the key enzymes and glucose transporters associated with glucose metabolism, such as GLUT1, are upregulated. The KRAS-driven MAPK pathway and transcription factor MYC are thought to be essential to the regulation process, although the underlying mechanism for the refined regulation of the related enzymes requires further investigation (8–10). In addition to the reprogramming of glycolysis, mutant KRAS signaling contributes to phosphoglycerate kinase 1 (PGK1) translocation in mitochondrion, leading to PDHK1 phosphorylation and Oxphos restriction in pancreatic cancer cells (11). Intriguingly, KRAS mutations are also induced as a consequence of glucose deprivation, indicating mutual interplay between the oncogene and metabolism (12). Furthermore, some overexpressed enzymes upregulated by KRAS, such as RPIA, remain unchanged in some pancreatic cancer cell lines with KRAS deletion to maintain non-oxidative pentose phosphate pathway (PPP) and cancer cell survival *via* a KRAS-independent pathway (13). Therefore, further investigation must be conducted to reveal the mutual regulation pathways between KRAS and metabolic reprogramming.

Given that the metabolic reprogramming process is closely related to tumor cell survival, progression, and immune evasion, reprogrammed metabolism may play a critical role in tumor microenvironment (TME) modulation in PDAC. First, the competitive uptake of glucose by tumor cells restricts immune cell activation, differentiation, and function by robbing immune cells of energy substances (14). Second, a mass of lactate produced by the aberrant glycolysis of tumor cells establishes an acidified TME, which favors tumor progression and immune suppression (15). Additionally, lactate directly impairs the immunosurveillance function of T cells (16, 17). Beyond immunomodulation, enhanced glucose metabolism also leads to resistance to gemcitabine-induced apoptosis of PDAC cells (18). Taken together, the reprogramming of glucose metabolism plays a pivotal role in PDAC progression and immune evasion.

Calcium- and integrin-binding protein 1 (CIB1), also known as calmyrin, is an intra-cellular Ca2+-binding protein with EF-hand domains (19). CIB1 has no enzymatic activity, but it has various binding partners, such as sphingosine kinase 1 (SK1), and is involved in a broad spectrum of cellular processes (20). SK1-expressing cells show a significant increase in glucose uptake and induction of aerobic glycolysis, affecting metabolic pathways related to the biosynthesis of macromolecules. Overexpression of CIB1 has been shown to correlate with oncogenic mutations of KRas, drive the localization of SK1 to the plasma membrane, and enhance the membrane-associated enzymatic activity of SK1 with its oncogenic signaling and is also related to glucose metabolism. CIB1 has also been confirmed to contribute to two common oncogenic pathways, PI3K/AKT and Ras/MEK/ERK, which are essential for tumor cell survival and proliferation (21–23). Here, we hypothesized that CIB1 may be associated with metabolic reprogramming in PDAC and may thus affect the TME *via* modulating tumor and stromal cellular components and immune cells to establish an immunosuppressive TME.

Metabolic reprogramming, the KRAS pathway, and relevant molecules may modulate the tumor, immune, and stromal components. Here, by analyzing these pathways, we identified that CIB1 was upregulated and associated with poor prognosis in patients with PDAC from The Cancer Genome Atlas (TCGA) database. We further identified that CIB1 was positively correlated with the glycolysis, oxidative phosphorylation (Oxphos), and hypoxia pathways, as well as the cell cycle, which reflected increasing numbers of tumor cellular components in the TME. The results of immunohistochemistry staining demonstrated that high expression of CIB1 in tumor cells was associated with increased tumor compartments and reduced stromal cellular abundance. Moreover, multiplexed immunohistochemistry (mIHC) verified that low stromal abundance also correlated with the low infiltration of CD8^+^PD-1^-^ T lymphocytes. These results reveal the linkage between CIB1, reprogrammed glucose metabolism, and the KRAS pathway, as well as the relevance of CIB1 expression to tumor and stromal cellular components and immune cell modulation in the TME.

## 2 Methods and materials

### 2.1 Data acquisition

The mRNA expression profile and the related clinical data were collected from the Gene Expression Omnibus (GEO) and TCGA databases. The selected samples met the following criteria: 1) an overall survival > 2 months; 2) complete survival status information; and 3) diagnosed with PDAC. Under these criteria, 116 samples from GSE183795, 109 samples from GSE71729, and 134 samples from TCGA were selected for further analysis. The accession number of murine mRNA expression profile is GSE127891.

The mRNA expression profile of pancreatic cancer cell lines and KRAS mutation information were obtained from the Cancer Cell Line Encyclopedia (CCLE) database (https://sites.broadinstitute.org/ccle/datasets, accessed on January 14, 2023),

Immunohistochemistry slice images of CIB1 in pancreatic cancers were obtained from the Human Protein Atlas (HPA) database.

The HALLMARK gene sets and Kyoto Encyclopedia of Genes and Genomes (KEGG) pathways gene sets were obtained from the GSEA Molecular Signature Database (https://www.gsea-msigdb.org/gsea/msigdb/inde x.jsp, accessed on January 14, 2023).

### 2.2 Prediction of immune cell infiltration

The prediction’ of immune cell infiltration was conducted *via* xCell (https://xcell.ucsf.edu/, accessed on September 22, 2022). A higher score estimated by the immune score or stromal score indicated a larger number of immune or matrix cellular components in the TME.

### 2.3 Survival analysis

The “Survival Analysis” module of GEPIA2 was used to generate survival plots with log-rank P-values. The survival plots for CIB1 in pancreatic adenocarcinoma in TCGA database were obtained *via* the GEPIA2 website. High- and low-expression CIB1 cohorts were obtained through the expression threshold of the cutoff-high (75%) and cutoff-low (25%) values. To validate the discovery in TCGA cohort, a survival test of CIB1 was also performed in GSE71729, with the median value of expression as the cutoff, *via* the R “survival” package. Survival analysis of the 8-gene signature was performed in GEPIA2 with the “Signatures” option in the “Survival Analysis” module.

### 2.4 Non-negative matrix factorization (NMF) and immunophenotype detection

NMF clustering was performed to virtually microdissect the immunophenotypes of PDAC based on the immune cell infiltration. The rank of clusters was determined based on cophenetic indicators, and the optimal clustering number was selected as 3.

### 2.5 Patient cohort and tissue microarray construction

Human PDAC specimens were obtained from Ruijin Hospital, Shanghai Jiao Tong University School of Medicine (Shanghai, China), with written informed consent from all participants. The study was approved by the Human Ethics Committees of Ruijin Hospital, Shanghai Jiao Tong University School of Medicine. The human tissue specimens were formalin-fixed and paraffin-embedded (FFPE). Based on the H&E staining results examined by a pathologist, cancer FFPE tissue samples from 35 patients were punched and arranged in tissue microarray (TMA) blocks. The clinical sample information of each patient is presented in **Supplementary Table 1**. The diameter of each block core used in this TMA assessment was 1.5 mm.

### 2.6 Multiplex IHC staining and image acquisition

We used the Ultivue UltiMapper Immuno 8 kit to conduct the mIHC staining according to the manufacturer’s instructions (#ULT20101, Ultivue, Cambridge, MA, USA) (24). Paraffin-embedded sections were heated in an oven set to 60°C for 1 h, deparaffinized with xylene, and rehydrated through a gradient of ethanol solutions. Antigen retrieval was performed in a pH 9 buffer, and antibody diluent was used to block the binding of nonspecific antibodies. The commercialized primary antibodies used were pre-designed panels for identifying specific cells in the TME and included anti-CD8α, anti-PD-1 and anti-pan-keratin antibodies. All antibodies were diluted in the ratio 1:100 in the antibody diluent and combined. The sections were then incubated for 1 h in the antibody mixture. After applying the Pre-Amplification Mix and the amplification enzyme solution to detect antibody staining, the tissues were incubated with Nuclear Counterstain solution, and the first-round fluorescent probe solution was used to detect CD8 and PD-1. A coverslip was then mounted over the tissue chip using ProLong Gold Antifade Mountant (Thermo Fisher, MA, United States). The sample was loaded onto the Vectra Polaris Automated Quantitative Pathology Imaging System (Akoya Biosciences, MA, United States), and whole slide scanning was employed to capture the first-round images at 20× magnification. After acquiring the first-round images, an exchange solution was used to remove the fluorescent probes. Subsequently, the second round of staining was conducted (pan-keratin), and images were captured as described above.

### 2.7 Image analysis

The HALO Image Analysis Platform (Indica Labs, v3.3.2541.345 was used for image overlay, tissue segmentation, and cell phenotype analysis. Positive thresholds for each marker were set based on the nuclear (DAPI) or cytoplasmic (CD8, PD-1 and pan-keratin) staining intensity and were examined across all tissue samples. Combined results for cell counts, densities, and percentages were exported for further analysis and generation of graphic images using a previously described method (25).

### 2.8 Gene set enrichment analysis (GSEA) and single sample gene set enrichment analysis (ssGSEA)

GSEA was performed with the R “clusterProfiler” package. The normalized enrichment score (NES) and adjust p value were used to quantify the enrichment magnitude and statistical significance, respectively. “GSVA” R package (26) (version 1.36.2) was applied to calculate ssGSEA scores of pathways and HALLMARK signatures in each sample. ssGSEA were performed with parameters as method = ‘ssgsea’, kcdf = ‘Poisson’.

### 2.9 Quantitative analysis of IHC slice images from HPA

The QuPath software (27) was downloaded and utilized to quantitatively measure the tumor cells and stromal cells of IHC slice images of CIB1 in patients with pancreatic cancer. 8 well stained slice images (4 slices with high expression of CIB1 and 4 slices with low expression of CIB1) were selected for the measurement. Those samples with strongly stained CIB1 were considered as with high expression of CIB1. The other samples were considered as with low expression of CIB1.

### 2.10 Cell culture

The KRAS^G12D^; Trp53^R172H^; Pdx1-Cre (KPC) cell lines were seeded (2 × 10^5/^well) in 12-well plates, cultured in DMEM (1000 μL). All the above mediums were supplemented with 10% FBS (Shanghai Life iLab Biotech Co., LTD), 100 U/mL penicillin, and 100 μg/mL streptomycin (Shanghai Life iLab Biotech Co., LTD).

### 2.11 Determination of glucose and lactate levels

The levels of glucose and lactate in supernatant of cell culture were determined by AU5800 (Beckman Coulter, Inc.) according to the manufacturer. The glucose consumption was calculated by the following formula.


$$Glucose\;consumption=\left(1-\frac{Glucose\;concentration\;of\;supernatant}{Glucose\;concentration\;of\;fresh\;DMEM\;\left(24.98\;mM\right)}\right)\times 100\%$$

### 2.12 siRNA interference of CIB1

CIB1 siRNA oligonucleotides and negative control siRNA were purchased from Shanghai GenePharma Co.,Ltd. siRNA 1: 5’-CCGCAUCUUUGACUUUGAU-3’. KPC cells were transfected with either CIB1 siRNA or negative control siRNA according to the manufacturer. The transfected cells were incubated for 48h before further experiments. After the 48h incubation, we replaced the medium was fresh medium and incubated the transfected cells for another 24h before we determined the glucose consumption and lactate production levels in supernatant.

### 2.13 Western blots

Anti-CIB1 polyclonal antibodies were purchased from Proteintech Group, Inc. Cells were lysed and proteins were separated by 12.5% SDS-PAGE, transferred onto PVDF membranes. Then the PVDF membrane was blocked with 5% non-fat milk and incubated with anti-CIB1 antibodies for 12h under 4 C. The secondary antibody was then incubated for 1h under room temperature. The bands were visualized and analyzed by ODYSEEY system and ImageJ software(version 2.9.0).

### 2.14 Statistical analyses

Data were analyzed *via* R software (version 4.2.2) and GraphPad Prism 8.0.2 software (GraphPad, Inc., San Diego, CA, USA). Two-tailed t-tests were used to determine statistically significant differences in unpaired data. One way ANOVA was used for multiple comparison. Pearson correlation tests were used to determine correlations in unpaired data. Survival analyses were performed to evaluate the prognostic value of the indices used in the study. Kaplan–Meier (K-M) analysis was used to plot survival curves *via* the log-rank test. P-values< 0.05 were considered significant.

## 3 Results

### 3.1 Calcium and integrin binding 1 (CIB1) was highly co-expressed with KRAS mutation and was related to metabolic reprogramming

**Figure 1:**
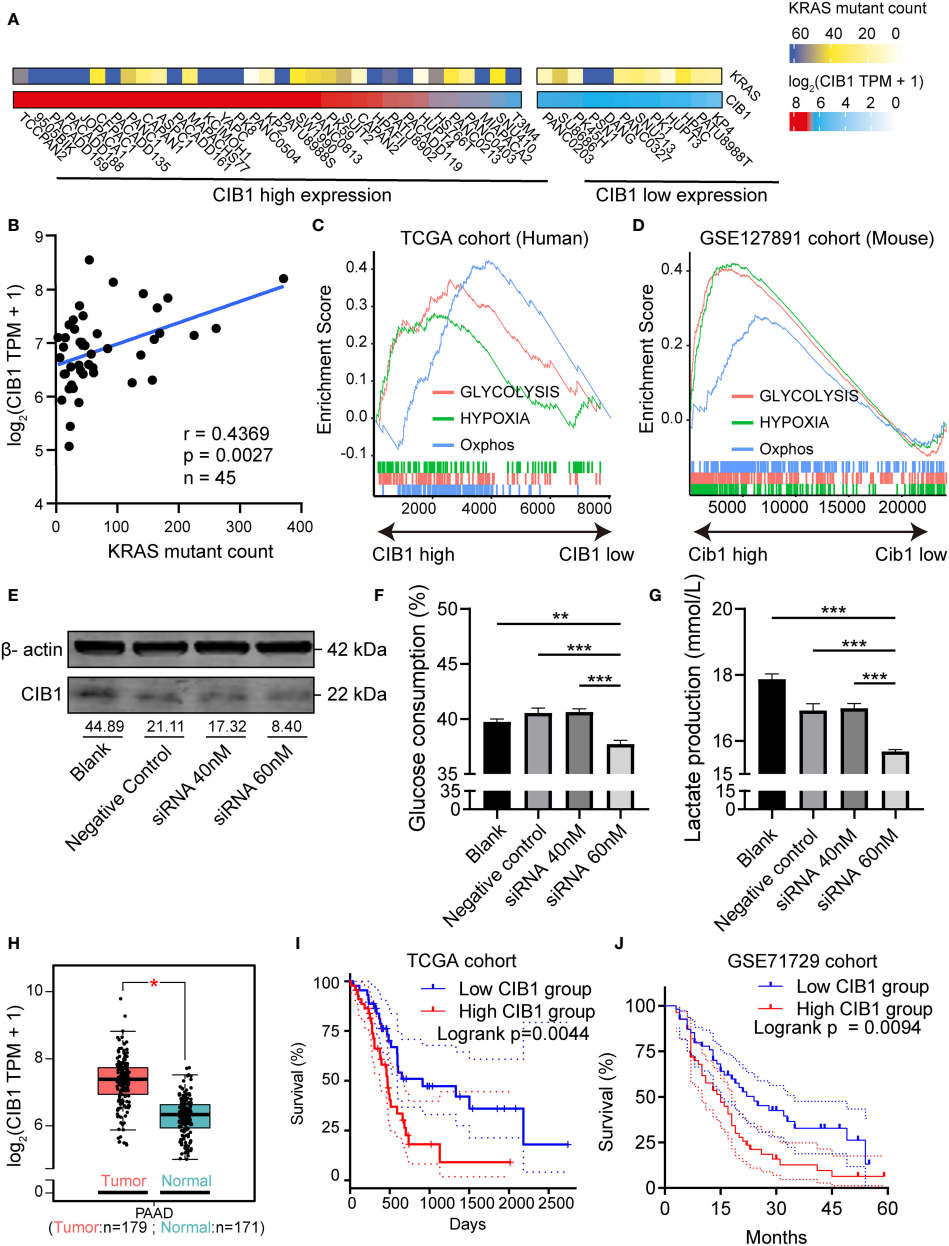
Calcium and Integrin Binding 1 (CIB1) was highly co-expressed with KRAS mutation and was related to metabolic reprogramming. **(A)** Alternative allele counts of KRAS in pancreatic cancer cell lines with different expression level of CIB1. The lower quartile of CIB1 expression in pancreatic cancer cell lines was used as the cutoff to determine the CIB1 expression levels. **(B)** Correlation scatter plots of alternative counts of KRAS and CIB1 expression. **(C)** Upregulated HALLMARK signatures in samples with high CIB1 expression. Glucose metabolism-related gene sets are represented as lines of unique color. Gene sets with p< 0.05 were considered significant, n = 134. **(D)** Upregulated HALLMARK signature in murine samples with high Cib1 expression,n = 8. **(E)** Western Blot validation of CIB1 knockdown *via* siRNA. The mean gray value of bands was measured by ImageJ. **(F)**Glucose consumption levels decreased under CIB1 knockdown condition. **(G)**Lactate production levels decreased under CIB1 knockdown condition. **(H)** Differential expression of CIB1 mRNA expression between pancreatic cancer and normal samples. **(I, J)** Survival plots of CIB1 in TCGA and GSE71729 datasets. Blue lines represent the survival curve of the low CIB1 expression group, and red lines represent the survival curve of the high CIB1 expression group. In TCGA cohort, n(high) = 45, n(low) = 45. In GSE71729 cohort, n(high) = 54, n(low) = 55 *p< 0.05. **p< 0.01, ***p< 0.001.

The mRNA profiles in pancreatic adenocarcinoma cell lines were obtained to confirm the expression of CIB1. The heatmaps showed that CIB1 is widely expressed in pancreatic adenocarcinoma cell lines, in which highly frequent KRAS mutation was also observed (**Figure 1A**). Further, the expression of CIB1 was positively correlated with the count of alternative allele of KRAS (**Figure 1B**). The co-expression of CIB1 and KRAS mutation suggests an interplay between CIB1 and downstream KRAS signaling.

Later, the GSEA pathway analysis was conducted to reveal the CIB1-mediated pathways. The results of pathway analysis demonstrated that hallmark signatures of glycolysis, hypoxia, and oxidative phosphorylation were upregulated in the high CIB1 group, indicating that glucose metabolism was reprogrammed and an elevated glucose metabolism under hypoxic condition corresponds to high CIB1 expression in both human and murine samples (**Figures 1C, D**). The full list of HALLMARK pathway analysis results was presented in **Supplementary Table 2**. Eight overlapped glucose metabolism-related genes were identified, all of which were demonstrated to be associated with low immune cell infiltration (**Supplementary Figures 1A–C**), indicating an immunosuppressive function of reprogrammed glucose metabolism.

In order to verify the linkage between KRAS mutation, CIB1 and glycolysis, we selected KPC cell line which contains KRAS^G12D^ mutation to conduct *in vitro* CIB1 knockdown *via* siRNA interference.The knockdown effect of siRNA was validated by western blot and the expression of CIB1 decreased when the concentration of siRNA increased (**Figure 1E**). Then we compared the glucose consumption and lactate production levels in supernatant of cell cultures and the results turned out that the glucose consumption and lactate production levels were significantly decreased, indicating a suppressed glycolysis under CIB1 knockdown condition (**Figures 1F, G**). As CIB1 expression was proven to be associated with KRAS mutation and reprogrammed glucose metabolism, a comparison of CIB1 expression between tumor and normal tissues in TCGA cohort revealed that CIB1 was upregulated in PDAC (**Figure 1H**). Further, survival tests revealed that patients with high CIB1 expression had lower OS than patients with low expression in TCGA cohort and GSE71729 cohort (**Figures 1I, J**, risk tables in **Supplementary Table 4**). Collectively, CIB1 was demonstrated to co-express with KRAS mutation, associated with elevated glucose metabolism and be of prognostic value in PDAC.

### 3.2 CIB1-related metabolism reprogramming suppressed immune cell infiltration in PDAC

**Figure 2:**
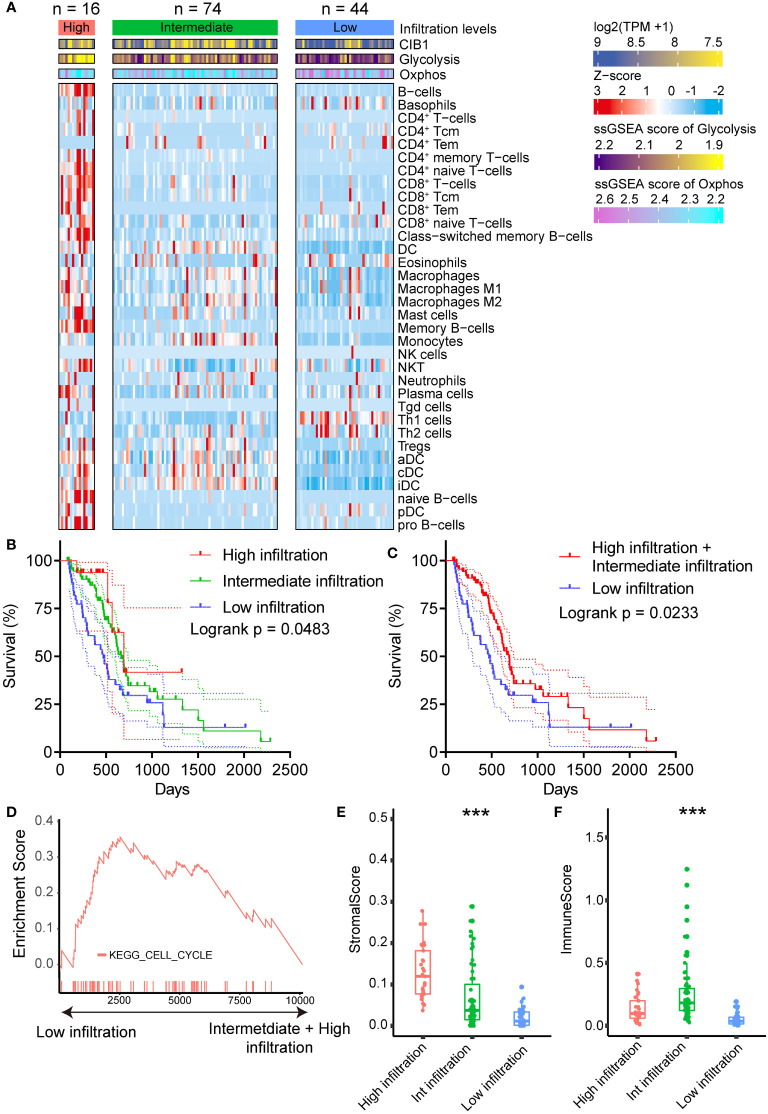
CIB1-related metabolism reprogramming suppressed immune cell infiltration in PDAC. **(A)** Heatmap representation of the three infiltration levels of immune cells in PDAC. The annotation bars indicated the clusters generated by NMF, CIB1 expression and ssGSEA score of Oxphos and Glycolysis. **(B, C)** Survival plots of patients with different immune cell infiltration levels in TCGA cohort. **(D)** Up-regulation KEGG pathways in samples with a low immune cell infiltration level. **(E)** Comparison of stromal scores between the infiltration levels of immune cells in PDAC. **(F)** Comparison of the immune score between patients with PDAC with three levels of infiltrating immune cells. *p< 0.05, **p< 0.01, ***p< 0.001.

After we confirmed the positive association between CIB1 expression and elevated glucose metabolism under hypoxic condition, we moved on to investigate the relationship between CIB1-related metabolism reprogramming and immune cell infiltration. To further portray the immune subtypes of PDAC, the xCell algorithm was used to predict the infiltration of immune cells, and the NMF algorithm was performed to virtually microdissect the immune subtypes of PDAC into three categories (high, intermediate, and low immune infiltration level) based on the infiltrating immune cell abundance (**Figure 2A**). The determination of rank used for clustering presented in **Supplementary Figures 2A, B**. In three categories, PDAC with low immune infiltration level was characterized by lowest infiltration of most immune cells but high expression of CIB1 and elevated glycolysis and Oxphos hallmark signature in comparison with the other two levels. As expected, compared to the other two levels, patients with low level of immune cell infiltration had the worst prognosis (**Figures 2B, C****, risk tables in** **Supplementary Table 4**).

Furthermore, upregulated cell cycle pathway was observed in PDAC with low immune infiltration levels (**Figure 2D**), indicating that CIB1-related metabolism reprogramming was negatively associated with the infiltration of immune cells in PDAC and promoted the growth of tumor cells. Full list of KEGG pathways analysis results was presented in **Supplementary Table 3**. Additionally, beyond the infiltration abundance of immune cell subtypes, stromal score and immune score were also observed to fall into three levels in line with the immune cell infiltration levels (**Figures 2E, F**). PDAC with low immune infiltration levels had a significantly lower stromal score and immune score than the other two levels, suggesting a potential modulation of stromal components in PDAC by CIB1-related metabolism.

### 3.3 High CIB1 expression was associated with low immune cell infiltration

**Figure 3:**
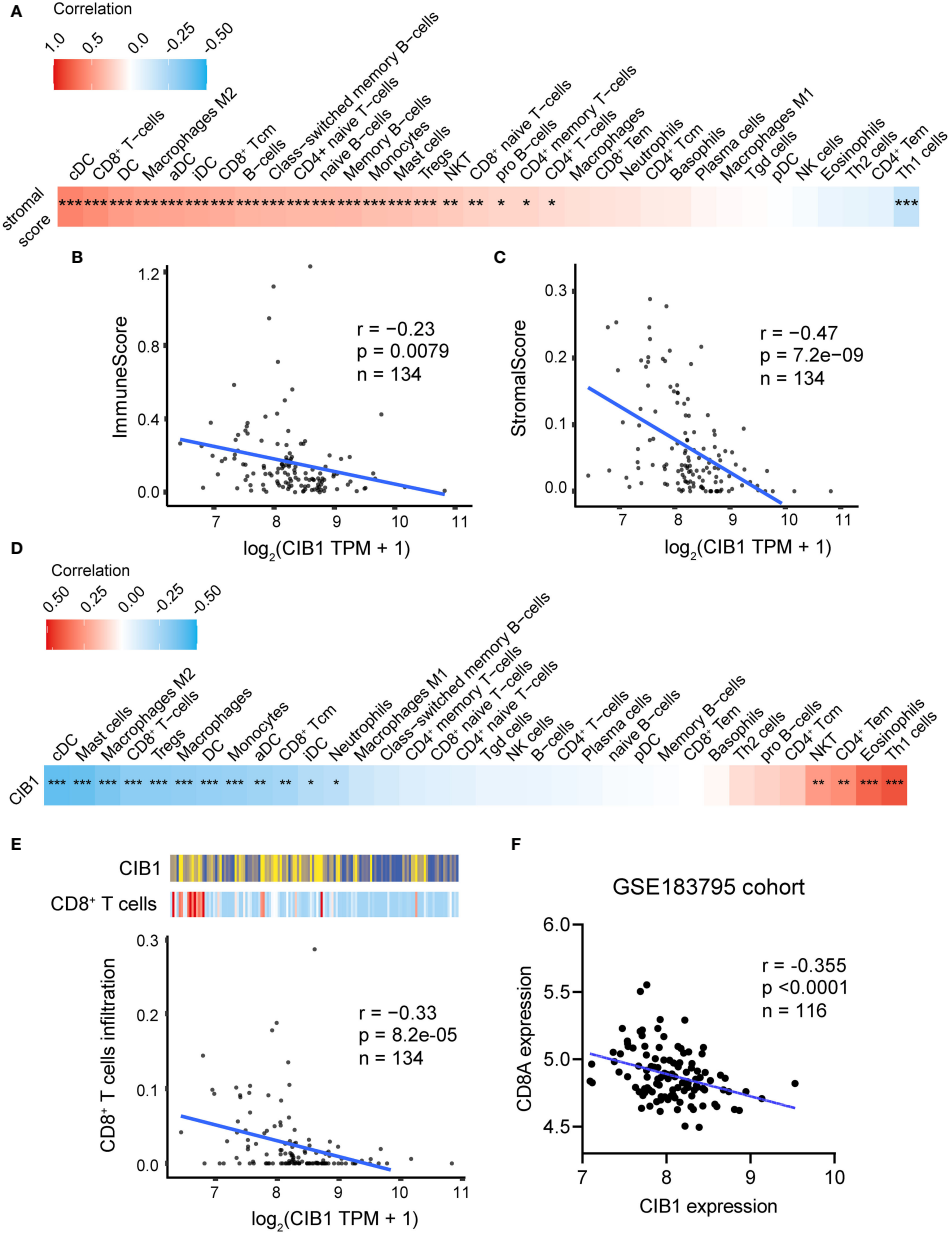
High expression of CIB1 was associated with low immune cell infiltration. **(A)** Correlation coefficients between stromal score and immune cell infiltration. Red represents a positive correlation, blue represents a negative correlation, and white represents no correlation. **(B)** Correlation scatterplots of CIB1 and immune score. **(C)** Correlation scatterplots of CIB1 and stromal score. **(D)** Correlation coefficients between CIB1expression and immune cell infiltration. **(E)** Correlation scatterplots of CIB1 and CD8^+^ T cells. **(F)** Correlation scatterplots of CIB1 expression and CD8A expression in GSE183795 cohrot, *p< 0.05, **p< 0.01, ***p< 0.001.

After confirming the association between CIB1, glucose metabolism, and immune infiltration levels, we considered that CIB1 itself was potentially immunosuppressive and modulated the stromal components in TME. The positive relationship between stromal score and infiltration of immune cell subtypes was confirmed, suggesting that abundant stromal components was in favor of immune cells infiltrating (**Figure 3A**). Beyond the infiltration abundance of a single immune cell subtype, CIB1 was negatively correlated with the immune and stroma scores, indicating that PDAC with high CIB1 expression had both low immune infiltration and low stromal cellular components (**Figures 3B, C**). Based on the immune cell infiltration estimated by xCell, we analyzed the correlation between CIB1 expression and immune cell infiltration, as shown in **Figures 3D, E**. The results all indicated that CIB1 was negatively associated with major anti-tumor contributors, notably, CD8^+^ T cells (**Figure 3E**) and high stromal cellular abundance was in favor of immune cell infiltrations. To validate the results, we assessed the correlation between expression of CD8+ T cells marker (CD8A) and expression of CIB1 in GSE183795 and the result was in line with the previous analysis (**Figures 3F**).

### 3.4 High tumor cell density was associated with low stromal cellular abundance in PDACs

**Figure 4:**
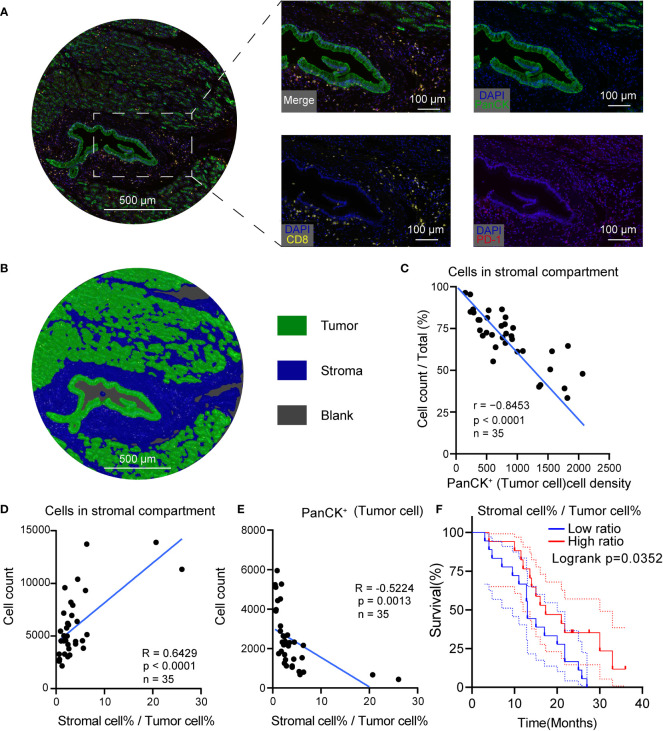
High tumor cell density was associated with low stromal cellular abundance in PDAC. **(A)** Representative mIHC images of PDAC. **(B)** Segmentation of the tumor tissue into tumor (green), stromal compartment (blue) and blank region (grey), Scale bar: 500 μm. **(C)** Correlation scatterplots of PanCK^+^ cell density and stromal cell %. PanCK^+^ cell density is the ratio of PanCK^+^ cell count and area of classified region. Stromal cell% is the ratio of count of cells in stromal compartment and total cell count. **(D)** Correlation scatterplots of stromal cell count and stromal cell%/tumor cell% Tumor cell %. Tumor cell % is the ratio of PanCK^+^ cell count and total cell count. **(E)** Correlation scatterplots of PanCK^+^ cell count and stromal cell%/tumor cell% **(F)** Survival plot of patients in high and low stromal/tumor groups. n(high ratio) = 17, n(low ratio) = 18.

To further measure the tumor, stroma, and immune cell components in PDAC tumor tissues, a workflow was established and optimized for mIHC to assess three markers (CD8, PD-1, PanCK) to simultaneously depict cell subtypes with single or multiple markers (**Figure 4A**). The PanCK marker was used to define the tumor cells, while DAPI was used to stain the nucleus of all cells. Further, specimens from patients with PDAC were prepared, fixed, and stained in a tissue microarray. Later, the Halo algorithm was used to identify and count the subpopulation of cell subtypes and segment the tumor tissues into tumor, stromal compartment and blank region (**Figure 4B**). The results of quantitative measurement showed that a high tumor cell density was related to a restricted stromal cell percentage (**Figure 4C**). The ratio of Stromal% and Tumor% (Stromal cell %/Tumor cell%) was used as an index to measure the relative structure of tumor cells and stromal cells. High stromal cell %/Tumor cell % indicated a TME compressing of abundant stromal cells and low tumor cells (**Figures 4D, E**). Beyond the structural relationship, the ratio of Stromal% and Tumor% were of prognostic significance (**Figure 4F****, risk tables in** **Supplementary Table 4**). High Stromal%/Tumor% indicated a favorable outcome.

### 3.5 PDAC with high CIB1 expression had more tumor components and fewer stromal components

**Figure 5:**
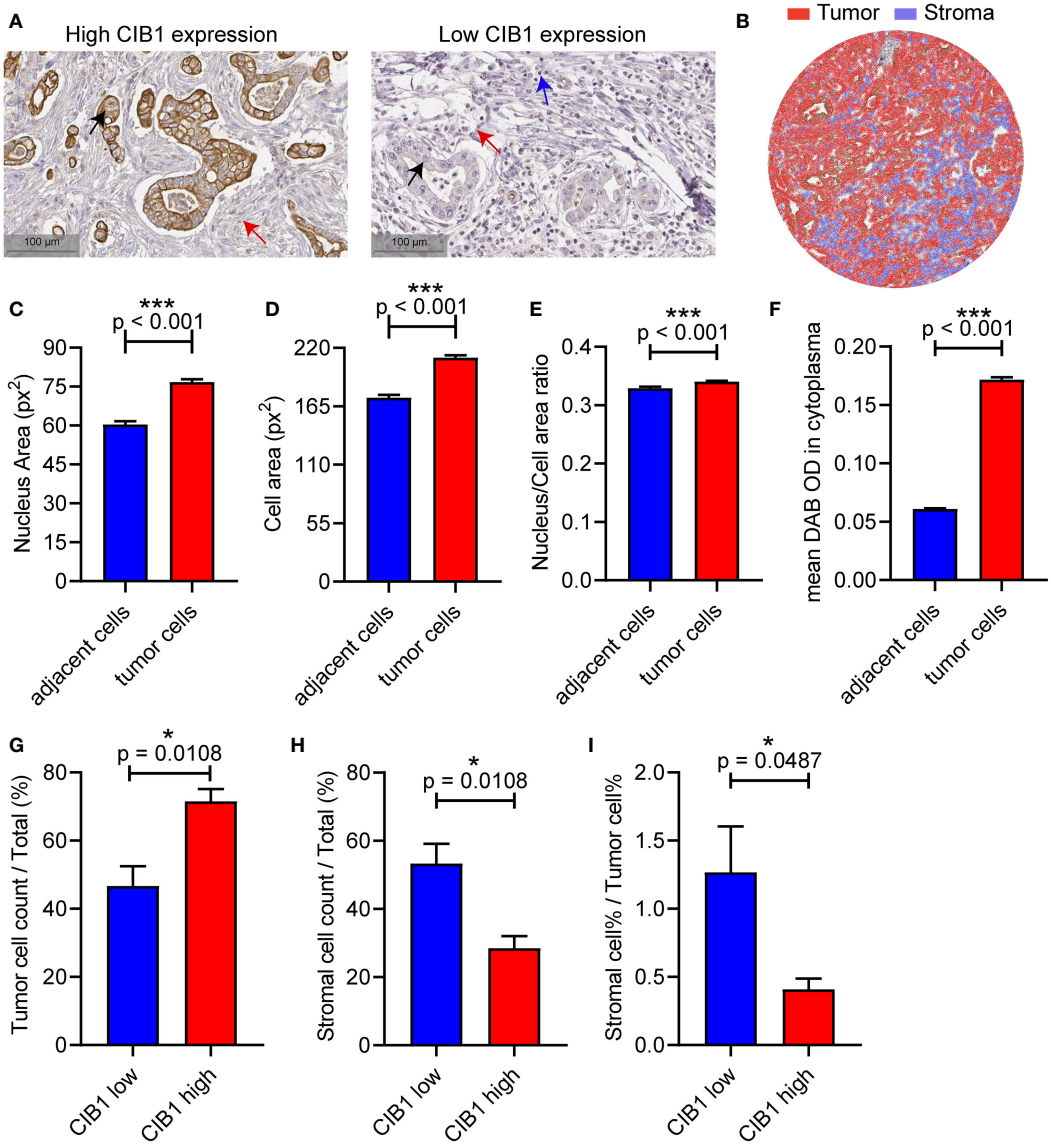
PDAC with high CIB1 expression was associated with low stromal cellular abundance and with low immune cell infiltration. **(A)** Representative IHC images of samples with high expression of CIB1 and those with low expression of CIB1. Black arrow: Cancer cell, red arrow: Fiber, blue arrow: Immune cell. **(B)** Illustration of quantitative analysis of IHC slice images. **(C-F)** Comparison of nucleus area, cell area, Nucleus/Cell area ratio and CIB1 expression between classified tumor cells and adjacent cells **(G-I)** Difference of tumor cell%, stromal cell%, and stromal cell %/tumor cell % ratio between samples with high expression of CIB1 and those with low expression of CIB1. *p< 0.05, Error bars represent the SEM.

To validate the relationship between CIB1 expression, tumor and stromal components in real world, we then obtained IHC images of CIB1 from the HPA database to further assess the association between CIB1 expression and the cellular components of the TME in PDAC. The IHC images of CIB1 in cancer samples suggested that samples with strongly stained CIB1 had fewer cells and were more fibrotic compared to CIB1-negative samples (**Figure 5A**), indicating that high expression of CIB1 was related to low stromal abundance. 8 well stained slice images (4 slices with high expression of CIB1 and 4 slices with low expression of CIB1) were selected and imported into QuPath software for quantitative measurement. The quantitative analysis, as illustrated in **Figure 5B**, was conducted to assess the tumor cells and stromal components difference between high and low CIB1 group. Those cells classified as tumors showed a higher nucleus area, cell area, Nucleus/Cell area ratio and CIB1 expression than those adjacent cells (**Figures 5C–F**). The results of quantitative analysis confirmed that high CIB1 expression was associated with fewer cells in stromal compartment and higher tumor components, compared with those with low CIB1 expression. (**Figures 5G, H**). Further, the ratio of stromal components and tumor components was significantly lower in samples with high CIB1 expression (**Figure 5I**), indicating that high CIB1 expression led to an unbalanced tumor-stromal structure with high tumor components but low stromal components. n(CIB1 high) = 4, n(CIB1 low) =4

### 3.6 A TME with abundant stromal components and low tumor cells was in favor of CD8^+^PD-1^−^ T cells infiltration in PDAC

**Figure 6:**
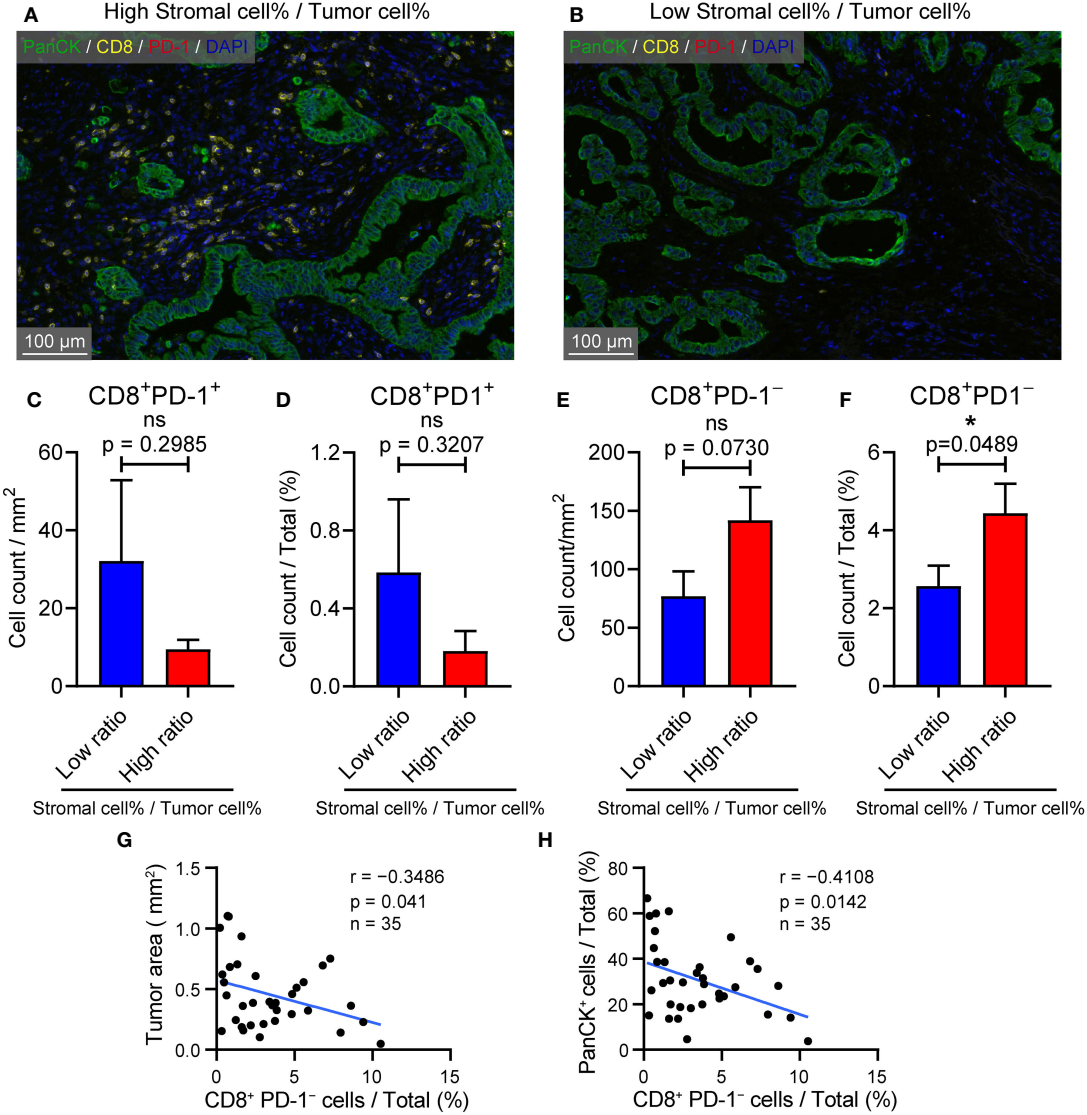
TME with high stromal and low tumor compartments is in favor of CD8^+^ PD-1^-^ T cell infiltration in PDAC. **(A)** Representative image of samples with abundant stromal cellular components and low tumor components. **(B)** Representative image of samples with low stromal cellular components and high tumor components. **(C)** Cell density of CD8^+^ PD-1^+^ T cells in samples with l low and high stromal cell %/tumor cell % ratio **(D)** CD8^+^ PD-1^+^ T cells % in samples with low and high stromal cell %/tumor cell % ratio. **(E)** Cell density of CD8^+^ PD-1^–^ T cells in samples with l low and high stromal cell %/tumor cell % ratio **(F)** CD8^+^ PD-1^–^ T cells % in samples with low and high stromal cell %/tumor cell % ratio. **(G)** Correlation between CD8^+^ PD-1^–^ T cells % and tumor area (mm^2^). **(H)** Correlation between CD8^+^ PD-1^–^ T cells % and PanCK^+^ %.

Previous results have confirmed that high CIB1 expression was associated with a TME compressing of high tumor components but low stromal components. The representative images of structure with abundant stromal cells and low tumor cells and structure with low stromal cells and high tumor cells were respectively presented in **Figures 6A, B**. To further assess the anti-tumor cells abundance in these two types of structures, we measured the count of CD8^+^PD-1^+^ and CD8^+^PD-1^−^ cells *via* Halo algorithm. The cell density and percentage of CD8^+^PD-1^+^ cells seemed to be more present in structure with low stromal cells and high tumor cells but was found to be of no statistically significant difference between two types of structures (**Figures 6C, D**).The structure with abundant stromal cells and low tumor cells had higher percentage of CD8^+^PD-1^−^ cells infiltration while the cell density of CD8^+^PD-1^−^ showed no difference. (**Figures 6E, F**). The higher percentage of CD8^+^PD-1^−^ cells infiltration was correlated with restricted tumor area and tumor components **(** **Figures 6G, H**). Collectively, a TME with high stromal components and low tumor cells is in favor of CD8^+^PD-1^−^ T cells infiltration in PDAC.

## 4 Discussion

Malignant cells, in complex with non-malignant cells, are an important component of tumors and form a heterogeneous TME. Non-malignant cells interact with malignant cells and contribute to the features of cancer. Non-malignant cells, which are the stromal cell components present in the TME, mainly include fibroblasts and immune cells such as lymphocytes and macrophages. Previous studies have highlighted the integral role of the TME in the progression of cancer (28–30). Mechanistically, the TME affects cancer cells through complex and dynamic pathways to modulate cancer-associated signaling, involving ligand-receptor interactions (e.g., PD-L1 binding of cancer cells to PD-1 of T cells), cytokines, metabolism reprogramming and KRAS pathways (31, 32). For instance, KRAS mutations which are widely detected in pancreatic cancers and colon cancers mediate multiple key glycolysis enzymes (such as HK-I/II, LDHA and GLUT1) thus leading glucose metabolism reprogramming (12, 13, 30, 32, 33). Among these pathways, we identified CIB1 as a key molecule related to metabolic and KRAS pathways. CIB1 was associated with increased tumor compartment and decreased stromal cell abundance, whereas low immune cell infiltration was associated with immunosuppression in the TME.

Along with the complex TME, extensive metabolic reprogramming, particularly reprogrammed glucose metabolism, also contributes to the immune evasion of PDAC cells under conditions of hypoxia and nutrient deprivation (13, 34). The reprogramming of glucose metabolism not only satisfies the requirements for energy and biosynthesis of essential cellular components but also facilitates the establishment of an immune suppressive microenvironment (35, 36). First, the competitive uptake of glucose by tumor cells restricts immune cell activation, differentiation, and function by robbing immune cells of the energy substance (14). Second, a mass of lactate produced by the aberrant glycolysis of tumor cells establishes an acidified TME, which favors tumor progression and immune suppression (15). Additionally, lactate directly impairs the immunosurveillance function of T cells (16, 17). Beyond immunomodulation, enhanced glucose metabolism also leads to resistance to gemcitabine-induced apoptosis of PDAC cells (18). Taken together, the reprogramming of glucose metabolism plays a pivotal role in PDAC progression and immune evasion.

Both TME components and reprogrammed glucose metabolism should be considered to assess the anti-tumor immune activity of PDAC. Hence, a biomarker that represents both will strengthen the ability to detect PDAC early and precisely. Here, we identified and verified CIB1 as being capable of predicting low stromal abundance and elevated glucose metabolism, which eventually led to an unfavorable prognosis.

CIB1 has been proven to be involved in the progression of triple-negative breast cancer and lung adenocarcinoma and has gradually been considered important in maintaining cell survival and proliferation (21, 22, 37). Further, elevated CIB1 has been reported to be associated with abnormal expression of oncogenic KRas and HRas, which are key drivers of metabolic reprogramming (23, 38). In this study, we found that CIB1 was widely co-expressed with KRAS mutation in pancreatic cancer cell lines and upregulated in tumor samples. The *in vitro* CIB1 knockdown validated the causality of CIB1 against glycolysis. Glycolysis was suppressed under CIB1 knockdown condition. The prognostic value of CIB1 was demonstrated in both TCGA cohort and GSE71729. High expression of CIB1 potentially serves as a marker for an unfavorable prognosis in PDAC. In line with previous studies, our data provide further evidence that CIB1 is associated with elevated glucose metabolism in PDAC. Eight overlapping genes involved in both glycolysis and Oxphos were demonstrated to be negatively associated with immune cell infiltration, especially CD8+ T cells, preliminarily verifying the hypothesis that CIB1 is associated with metabolic reprogramming in PDAC, leading to an immunosuppressive TME.

We conducted unsupervised clustering to virtually microdissect PDAC, with the aim to precisely portray the immune subtypes of PDAC. PDACs were subsequently divided into subtypes with three immune infiltration levels: high, intermediate, and low. The PDAC with low immune infiltration levels showed the highest CIB1 expression, the lowest immune cell infiltration, and the worst prognosis compared to the other two subtypes. Furthermore, elevated cell cycle and Oxphos pathways were found in the PDAC with low immune cell infiltration, indicating that reprogrammed glucose metabolism under hypoxic conditions contributes to a repressed anti-tumor immunity and tumor growth.

Given the positive association between CIB1 expression and elevated metabolic re-programming in PDAC, we next investigated whether CIB1 could serve as an indicator of the repressed immune landscape in PDAC. The results of correlation tests revealed that CIB1 was associated with an immunosuppressive landscape with reduced anti-tumor cell infiltration. Therefore, we can conclude that CIB1 is a biomarker for elevated glucose metabolism and immunosuppressive landscapes.

Intriguingly, our data also suggested that CIB1 expression was related to low stromal cellular component abundance in PDAC. The IHC images revealed that tumors with high CIB1 expression had a stromal region with fewer cellular components but a more fibrotic structure. Further, quantitative analysis of TMA data revealed the infiltration of fewer CD8^+^ PD-1^−^ T cells in PDAC with low stromal abundance. We confirmed that high CIB1 expression is associated with a low stromal cellular component abundance and low immune cell infiltration in PDAC, although the underlying mechanism requires further investigation. Together with immune cells, cancer-associated fibroblasts (CAFs) are considered an important and heterogenic component of PDAC. In a previous study, Yu Wang et al. identified a unique subtype of CAFs with a highly activated metabolic state (meCAFs) in PDAC (39). The high abundance of meCAFs will lead to an unfavorable prognosis and a high risk of metastasis. In this study, due to the limitation of staining panels, the relationship between CAFs and immune cell infiltration was not tested and validated. Therefore, further studies are necessary to explore the interplay between CAFs and immune cells. Other limitations still existed in this study. Although we validated the causality of CIB1 against glycolysis, the mechanism underlying how CIB1 interact with participants in glycolysis and alter the cell metabolism was unclear. On the other hand, whether the knockdown of CIB1 would eventually boost the anti-tumor immunity was unknown.

In conclusion, we systematically characterized CIB1 as the key molecule linked to metabolic and KRAS pathways. CIB1 not only has a possible relationship with metabolic reprogramming in glycolysis, Oxphos, and hypoxia pathways but may also be associated with low immune cell infiltration in the stromal compartment of PDAC for re-programmed glucose metabolism and immune modulation, highlighting CIB1 as a predictive biomarker for prognosis.

## Data availability statement

The original contributions presented in the study are included in the article/**Supplementary Material**. Further inquiries can be directed to the corresponding authors.

## Ethics statement

The studies involving human participants were reviewed and approved by Shanghai Jiao Tong University Human Ethics Committees. The patients/participants provided their written informed consent to participate in this study.

## Author contributions

DX and JM contributed to the concept and designed the research work. JM, YS, TZ, XY, HY performed the experiments, acquisition, analysis and interpretation of the data. JM, YS, TZ, JD, YH and DX drafted the article. JZ, JL, YX, XF, DWC critically revised for important intellectual content. PX, PN, DW, JD, DX and YH provided critical reagents and supervised the research. All authors contributed to the article and approved the submitted version.

## Supplementary material

Supplementary Table 1 clinical_information.xlsx.

Supplementary Table 2 HALLMARK signatures list.xlsx.

Supplementary Table 3 KEGG pathway list.xlsx.

Supplementary Table 4 Risk tables of survival plots.xlsx.
