# Supplementary figures and images for: Identification of calcium and integrin-binding protein 1 as a reprogrammed glucose metabolism mediator to restrict immune cell infiltration in the stromal compartment of pancreatic ductal adenocarcinoma
Source: Front Immunol. 2023 Apr 28;14:1158964. doi: 10.3389/fimmu.2023.1158964 (PMC10175692; doi:10.3389/fimmu.2023.1158964)

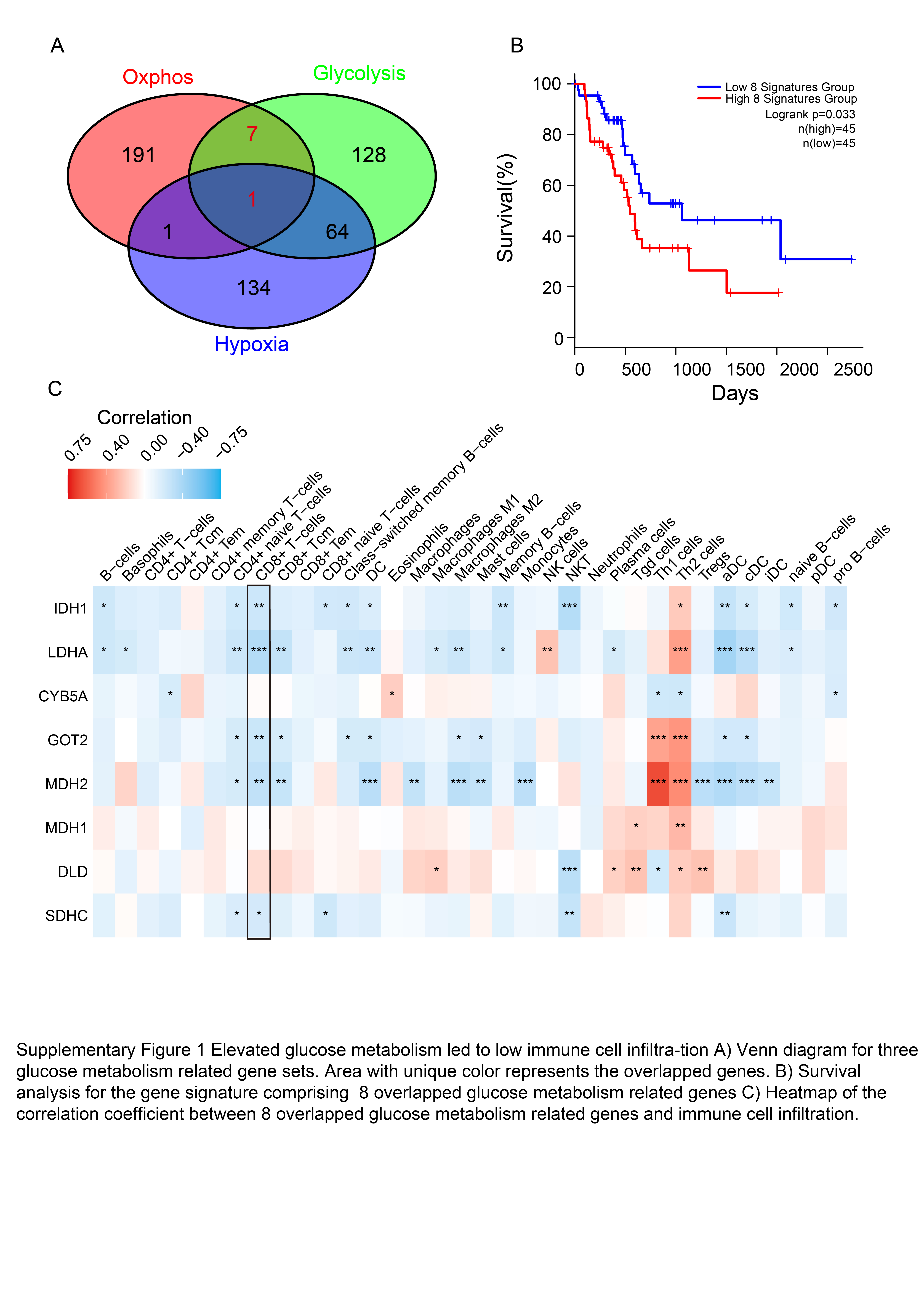

Supplement: Supplementary file 5 [file Image_1.jpg]

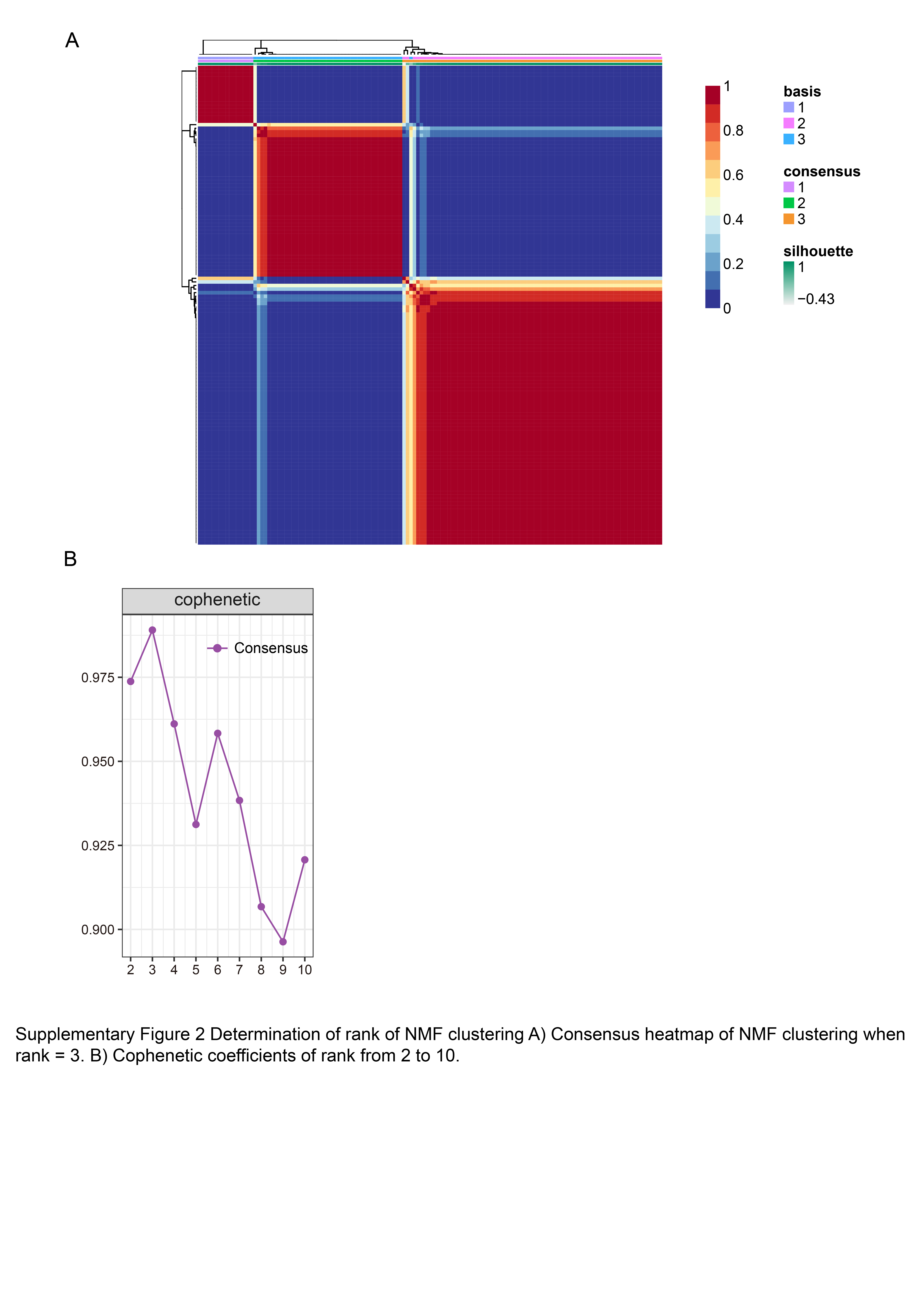

Supplement: Supplementary file 6 [file Image_2.jpg]
